# Future climate warming and changes to mountain permafrost in the Bolivian Andes

**DOI:** 10.1007/s10584-016-1655-8

**Published:** 2016-04-13

**Authors:** Sally Rangecroft, Andrew J. Suggitt, Karen Anderson, Stephan Harrison

**Affiliations:** 1 Environment and Sustainability Institute, College of Life and Environmental Sciences, University of Exeter, Penryn Campus, Penryn, TR10 9FE UK; 2 Department of Geography, University of Exeter, Penryn Campus, Penryn, TR10 9FE UK

**Keywords:** Climate Change Adaptation, Water Security, Rock Glacier, Glacial Lake Outburst Flood, Glacier Recession

## Abstract

Water resources in many of the world’s arid mountain ranges are threatened by climate change, and in parts of the South American Andes this is exacerbated by glacier recession and population growth. Alternative sources of water, such as more resilient permafrost features (e.g. rock glaciers), are expected to become increasingly important as current warming continues. Assessments of current and future permafrost extent under climate change are not available for the Southern Hemisphere, yet are required to inform decision making over future water supply and climate change adaptation strategies. Here, downscaled model outputs were used to calculate the projected changes in permafrost extent for a first-order assessment of an example region, the Bolivian Andes. Using the 0 °C mean annual air temperature as a proxy for permafrost extent, these projections show that permafrost areas will shrink from present day extent by up to 95 % under warming projected for the 2050s and by 99 % for the 2080s (under the IPCC A1B scenario, given equilibrium conditions). Using active rock glaciers as a proxy for the lower limit of permafrost extent, we also estimate that projected temperature changes would drive a near total loss of currently active rock glaciers in this region by the end of the century. In conjunction with glacier recession, a loss of permafrost extent of this magnitude represents a water security problem for the latter part of the 21st century, and it is likely that this will have negative effects on one of South America’s fastest growing cities (La Paz), with similar implications for other arid mountain regions.

## Introduction

Water security in many arid mountain regions is under threat from climate change, glacier recession and population growth (Viviroli et al. 2011; Buytaert and De Bièvre 2012). With 99 % of the world’s tropical glaciers, the South American Andes are experiencing widespread and continuing glacier recession (Soruco et al. 2009; IPCC 2013). Components of the mountain cryosphere such as snow, glaciers, permafrost and thawing permafrost are especially sensitive to temperature changes because of their close proximity to melting and thawing conditions (Haeberli and Beniston 1998; Kääb et al. 2007). Here, the cryosphere acts as an important hydrological buffer, providing reliable stores of water for tens of millions of people, yet future security of supply for regions reliant on these features cannot be guaranteed.

Permafrost is defined as ground which remains at or below 0 °C for at least two consecutive years (Harris et al. 2003). Although the environmental conditions suitable for permafrost exist in most mountainous regions of sufficient elevation (Travassos et al. 2008; Viviroli et al. 2011), present understanding of Andean permafrost distribution and resilience is limited (Azócar and Brenning 2010). Most mountain regions have warmed faster than the global average (Bradley et al. 2006); for instance, warming in the European Alps since the 1980s has been reported as 0.5 °C per decade (EEA 2009). The Andes warmed at 0.11 °C per decade in the latter part of the 20th century, which is 0.06 °C per decade above the global average (Vuille et al. 2008). Continued warming is expected to cause further retreat and degradation of high-elevation permafrost (Haeberli et al. 1993). However, although it is “*virtually certain*” that Northern Hemisphere permafrost will continue to decline during the 21st century (IPCC 2013, p.1032), similar predictions for Southern Hemisphere permafrost are not available. This represents a substantial knowledge gap, particularly in regions where water security is already at risk. Here, we address this gap by providing a first-order assessment of the impact of climate change on mountain permafrost in the Bolivian Andes. We have used the Bolivian Andes as an example of an arid high mountain system to explore the vulnerability of these water stores to future climatic change, and to highlight the climate change adaptation issues that follow.

At local scales, the location of mountain permafrost is controlled by topographic and site-specific variables, such as level of snow cover, the slope and aspect of the surface, and its vegetation type and cover. However, at regional scales it is strongly correlated with the Mean Annual Air Temperature (MAAT), with the 0 °C isotherm often used to mark the lower elevational boundary (Del Barrio et al. 1990; Avian and Kellerer-Pirklbauer 2012). Although it is expected that atmospheric warming will cause an upward shift in this lowest elevation boundary (Haeberli et al. 1993; Janke 2005; Bonnaventure and Lewkowicz 2011), the coarse spatial resolution of current Global Climate Models (GCMs) does not permit a precise understanding of likely changes to future permafrost extent at the regional level, thereby preventing water resource managers adapting their policies to climate change (Hijmans et al. 2005; Buytaert et al. 2010). Downscaling these climate projections to an appropriate spatial resolution is therefore a necessary first step towards understanding climatic impacts of future global warming on permafrost water stores at the regional scale (Marengo et al. 2010), after which appropriate adaptation policies can be formulated.

Our study also examines the likely effect of climate change on active rock glaciers, which are a useful indicator of the lower boundary of mountain permafrost (Barsch 1996). These features consist of frozen rock debris and 40–60 % ice, and typically occur in high mountainous terrain (Barsch 1996; Brenning 2005). They are already considered to be important sources of water in montane, arid environments like the Chilean and Argentinean Andes (Croce and Milana 2002; Brenning 2005; Azócar and Brenning 2010), and may become more so as glaciers recede (Millar and Westfall 2008; Esper Angillieri 2009). While work modelling the implications of climate change on rock glaciers is limited, active rock glaciers have been used for modelling permafrost extents and changes in the North American Rocky Mountains (Janke 2005).

Here, we assess the impact of future warming on rock glaciers and permafrost extent in the arid Bolivian Andes by estimating changes in the distributions of land falling below thresholds for permafrost suitability (close to the frequently cited 0 °C isotherm). We adopt two approaches: first, we first use present day climate data (Hijmans et al. 2005) and statistically downscaled climate projections (Mitchell and Osborn 2005) to model present and future climates, enabling a first-order assessment of the effect of 21st century projected warming on permafrost extent. Second, we apply these climate data to a recent rock glacier inventory for the Bolivian Andes (Rangecroft et al. 2014), to model potential changes in activity over this period. Both our approaches suggest a marked decline in the area suitable for permafrost (and thus permafrost features) under climate change, potentially exacerbating the existing water scarcity problems in the Bolivian Andes. Other arid mountain regions dependent on cryospheric water supplies are likely to face similar problems this century.

## Study region

**Fig. 1:**
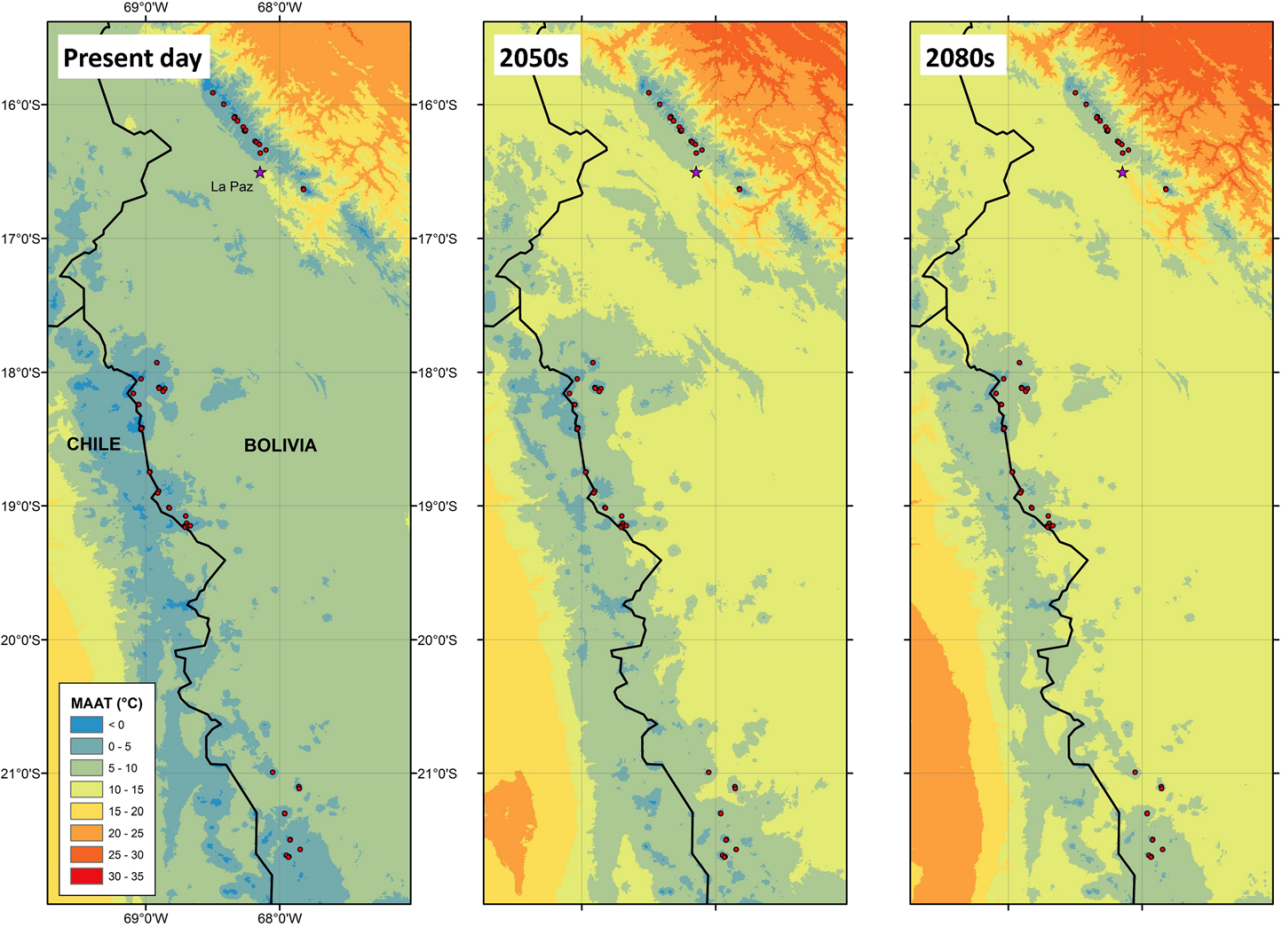
Present and future Mean Annual Air Temperatures (MAATs) for the study region. Present day (1950–2000) MAATs using WorldClim data (left panel). Multimodel ensemble mean projected MAAT from 7 downscaled GCMs for the IPCC A1B scenario for South America, generated from 2050s (middle) and 2080s (right) warming data. Figure was generated in ArcGIS 10.1, data with permission from ClimGen

The study region covers the two Cordillera mountain ranges of Bolivia between 15° S and 22° S (Fig. 1), divided into three climatically and topographically distinct regions: i) ‘Cordillera Real’ (15° - 16° S), this glaciated mountain region close to La Paz contains the highest density of glaciers in the country and has the wettest climate of the Bolivian Andes (~700 mm annually); ii) ‘Sajama’ (17° - 18° S), this region contains the isolated ice capped volcanic mountains in the Sajama National Park close to the Bolivia-Chile border; and iii) ‘Western Cordillera’ (18° - 22° S), this region is comprised of the dry, barren mountain range of the Cordillera Occidental (south of Sajama, see Rangecroft et al. 2014). Aridity increases towards the south, with the Western Cordillera receiving on average less than 200 mm annually (Jeschke 2009), resulting in no ice glaciers in the south (Jordan 1998). Overall, Bolivia has a distinctive climate consisting of a dry season (May – August) and a wet season (December – February). Temperatures and incident solar radiation are almost homogenous throughout the year, with temperatures 1 or 2 °C higher during the wet summer (Rabatel et al. 2013).

In the Bolivian Andes, nearly 50 % of glacier ice cover has been lost since 1960 (Soruco et al. 2009) and small and low-lying glaciers are projected to disappear within the next 20 years (Bradley et al. 2006; Vuille et al. 2008). Water supply deficiencies are particularly acute in the dry season, when the region is reliant on meltwater for domestic use, agriculture and energy generation. It is estimated that glacial melt water provides 12–40 % of the potable water for the Bolivian capital city, La Paz (Rangecroft et al. 2013). Despite this vulnerability in Bolivia, and across South America as a whole, there has been no previous research examining the implications of projected warming for the continent’s mountain permafrost.

## Methods

### Present day MAAT

Present day (1950–2000) MAAT estimates for the Bolivian Andes were derived from the ‘Worldclim’ monthly mean temperature data at 30 arc sec (~ 1 km) horizontal resolution (Hijmans et al. 2005, Fig. 1). The Worldclim data were gathered from global weather stations (Hijmans et al. 2005) and interpolated using a thin plated smoothing algorithm in ANUSPLIN (Hijmans et al. 2005). These data have been used extensively (Hole et al. 2009; Loarie et al. 2009) in the climate impacts literature due to their fine spatial resolution. The Bolivian Andes were defined as land above 3500 m above sea level within the political boundary of Bolivia. This successfully isolated the mountain areas, yet exclude the Bolivian tropics where permafrost is not found.

### Projected changes in MAAT and the proportion of land area suitable for permafrost

Projected future changes in temperature were derived from a downscaled multi-model ensemble of seven IPCC GCMs driven by the SRES A1B emissions scenario (Mitchell and Osborn 2005). This A1B scenario represents a future world of rapid economic and population growth, peaking mid-century (IPCC 2000). The multi-model ensemble dataset was constructed from seven GCMs by the ClimGen project (Mitchell and Osborn 2005). The ClimGen project downscaled the GCM data at 5° horizontal resolution to 0.5° resolution (~50 km in the Tropics) using the ‘pattern scaling’ approach. The projected changes in temperature were ‘draped’ over the present day data to generate estimates of projected future MAAT, for each GCM in the ensemble (*n* = 7 GCMs). We assessed the warming data associated with two ‘epochs’, or time periods: the 2050s (representing the time period 2040–2069) and 2080s (representing 2070–2099).

The projected future MAAT surfaces were used to assess the change in permafrost extent under equilibrium conditions. We classified land area as being suitable for permafrost if the temperature remained below specific MAAT thresholds. Because upper thresholds for permafrost suitability are reported to vary in different regions of the globe, we tested the sensitivity of our estimates to a range of seven thresholds (MAAT of −4 to +2 °C). Although the extent of permafrost is often marked by the 0 °C isotherm (Avian and Kellerer-Pirklbauer 2012), conservative thresholds of +1 and +2 °C were also used, to accommodate for any local topographic and microclimatic effects (Payne 1998). Colder thresholds for MAAT (closer to those reported in the Northern Hemisphere, e.g. Brenning 2005) were also tested.

### Projected changes to rock glacier activity

We adopted a Bolivian rock glacier inventory derived from a hybrid satellite and field mapping approach (cf. Rangecroft et al. 2014), using the criteria of Baroni et al. (2004). A total of 54 active rock glaciers in Bolivia were identified. The MAAT at the centroid of each active rock glacier was calculated for present day and future (2050s, 2080s) temperatures, using the data derived from each GCM (*n* = 7), and the ensemble mean. The number of active rock glaciers under 2050s and 2080s warming was estimated using seven upper thresholds (−4 to +2 °C) for activity.

## Results

### Projected temperature increase

Across South America, MAATs are projected to increase by a range of 0.8–3.4 °C by the 2050s and a range of 1.4–5.1 °C by the 2080s, relative to present day conditions (Fig. 1). Levels of warming projected for the Bolivian Andes were at the higher end of these ranges, with projections suggesting a 2.7–3.2 °C increase by the 2050s and 4.2–4.9 °C by the 2080s (Fig. 1b,c).

### Current and projected permafrost extent

**Fig. 2:**
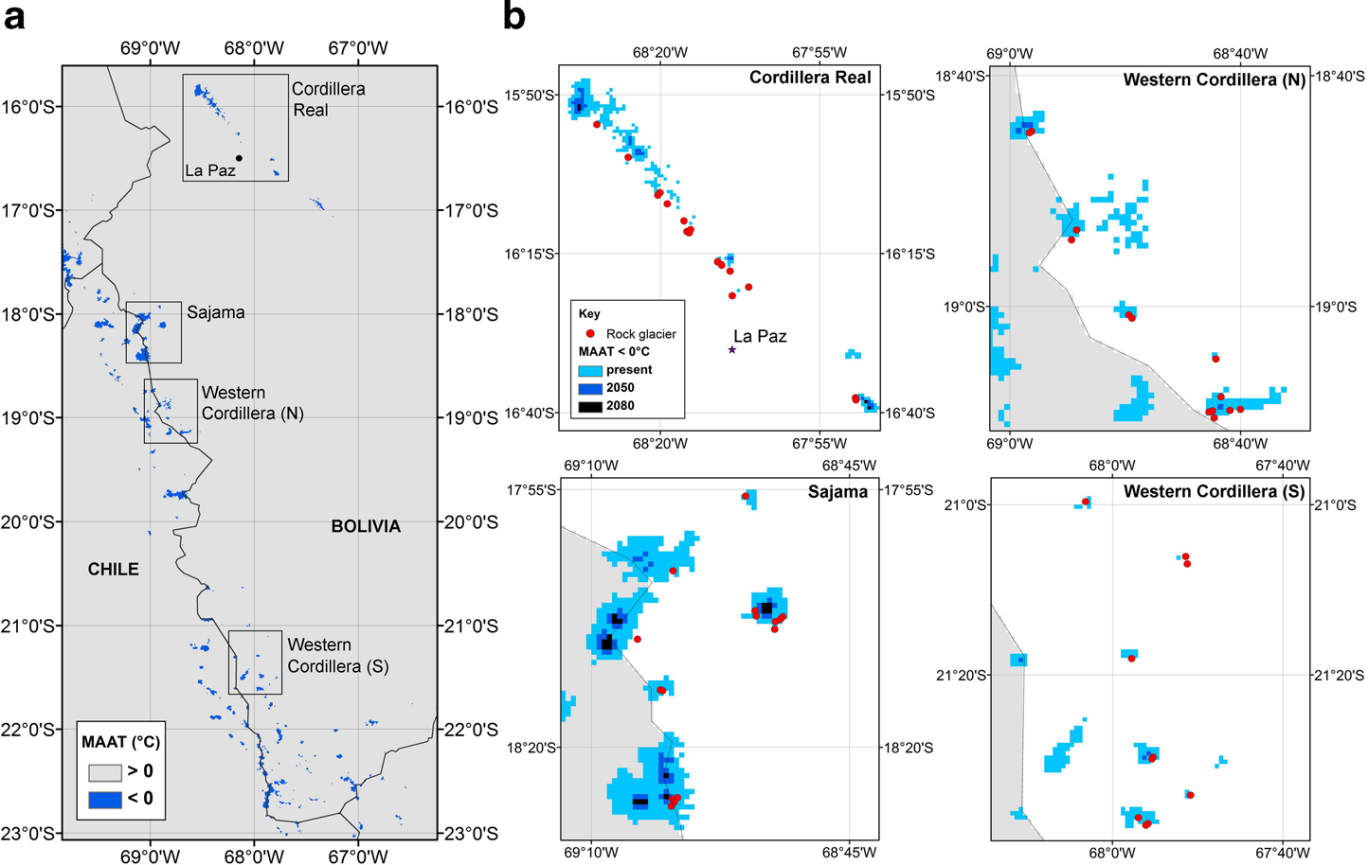
Climate change will reduce the area of land suitable for permafrost. Maps illustrate areas of present day and future projected Mean Annual Air Temperature (MAAT) below a suitability threshold of 0 °C, as a proxy for permafrost extent. **a)** Present day land area below a suitability threshold of 0 °C (blue coloration). **b)** Shrinkage of land area below a threshold MAAT of 0 °C in four example landscapes where rock glaciers are present in the Bolivian Andes. Figures were generated in ArcGIS 10.1

We used the 0 °C MAAT threshold to illustrate the extent of conditions suitable for permafrost under present day conditions (1950–2000), and the likely changes to this extent arising from 21st century projected warming (Fig. 2). Even under current climate conditions, permafrost is seen to occupy a fragmented “island” distribution across the Bolivian Andes (Fig. 2a). The area of suitable permafrost (under 0 °C MAAT) was projected to decrease by approximately 95 % under 2050s warming and 99 % under 2080s warming (Fig. 2b, Table 1). The size of these reductions was largely insensitive to the choice of MAAT threshold; projected reductions in extent based on alternative plausible thresholds (−4 to +2 °C) differed by less than 3 % for 2050s warming and less than 1 % for 2080s warming (Table 1).

**Table 1:** The percentage of Bolivian land area above 3500 m suitable for permafrost in the present day (1950–2000), changes in this area under projected climate warming and estimated number of rock glaciers remaining active given this warming. Seven isothermic thresholds for permafrost suitability were applied (MAAT range − 4 to +2 °C). The loss in land area under each of these thresholds due to future climate warming was calculated, based on the ensemble mean (in bold) and the range across models (in brackets). The number of rock glaciers estimated to remain active under each isothermic threshold for the ensemble mean are also shown for 2050s and 2080s warming. Values for the commonly cited MAAT threshold of 0 °C are shaded

Isothermic threshold	% Land area below threshold (present day)	% Loss in land area below threshold due to warming (ensemble range)		Number of rock glaciers estimated to remain active	
		With 2050s warming	With 2080s warming	With 2050s warming	With 2080s warming
< −4 °C	7.3 × 10^−3^	98.5 (94.7–100.0)	100.0 (100.0)	0	0
< −3 °C	2.6 × 10^−2^	96.0 (94.0–98.5)	100.0 (100.0)	0	0
< −2 °C	7.1 × 10^−2^	96.7 (95.1–97.3)	99.6 (98.4–100.0)	0	0
< −1 °C	2.2 × 10^−1^	95.9 (93.5–97.4)	99.3 (99.0–99.5)	0	0
< 0 °C	5.6 × 10^−1^	95.0 (92.7–96.5)	99.4 (98.2–99.6)	0	0
< +1 °C	1.4	94.2 (91.4–95.9)	98.9 (98.0–99.3)	3	0
< +2 °C	3.0	92.1 (88.8–94.5)	98.5 (97.5–99.0)	17	1

### Current and projected rock glacier temperatures

**Fig. 3:**
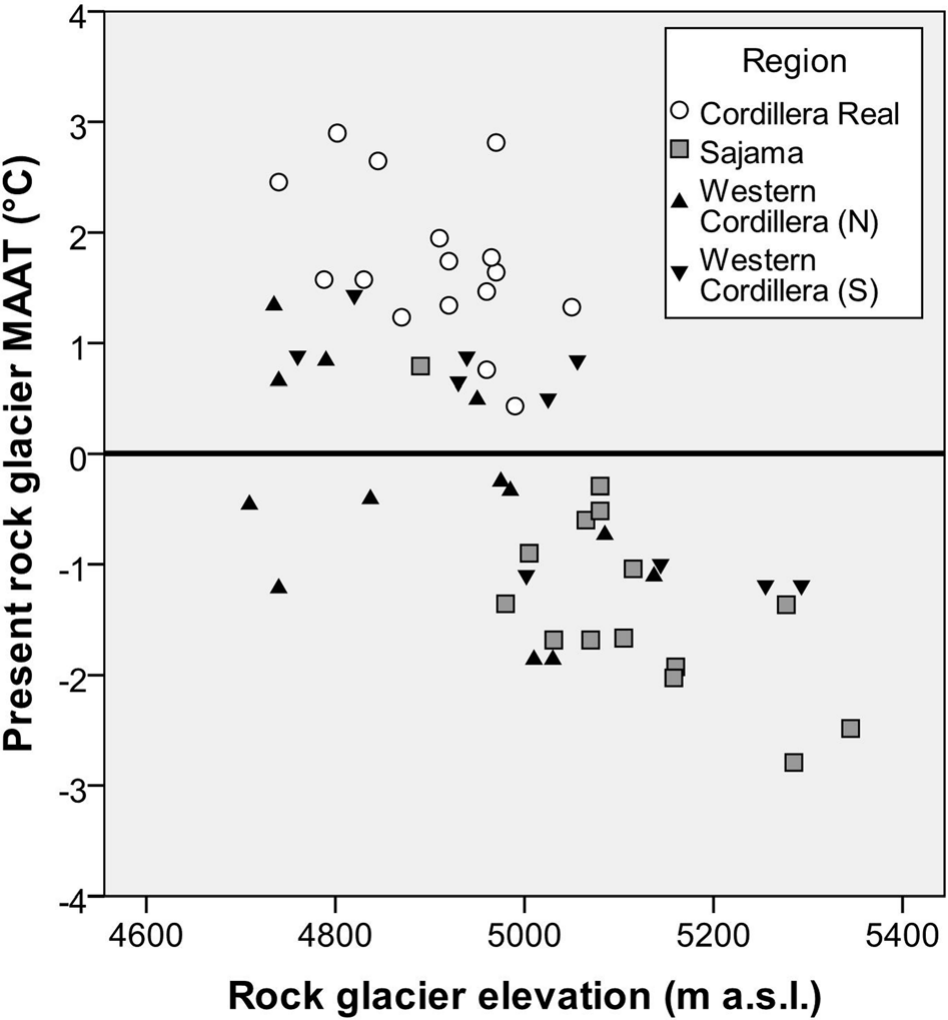
The present day MAAT at active rock glacier sites in the Bolivian Andes, plotted against site elevation. Figure was generated in SPSS

Active rock glacier sites have a present day average MAAT of +0.1 °C (95 % CI of ±0.4 °C) with a range of −2.8 to +2.9 °C across the 7 models used. Active rock glaciers were mostly clustered around the 0 °C threshold (Fig. 3), however differences were observed across the three different regions, with higher MAATs in the Cordillera Real than the other two regions (Fig. 3).

We found that the thermal conditions necessary for the persistence of active rock glaciers will deteriorate under future warming (Fig. 2b). Using future temperature projections at the rock glacier sites, we estimated that all currently active rock glaciers in Bolivia are projected to have an MAAT of more than 0 °C under warming levels projected for the 2050s. It must be emphasised that any change in the activity of a rock glacier as a result of this warming may lag behind the modelled temperature shift; this estimate therefore represents the final, equilibrium change in activity that could result. At present, some 93 % (50 of 54) active rock glaciers in the inventory lie within the more conservative, +2 °C activity threshold. However, under 2050s warming, the MAAT for 34 % (*n* = 17, range 10 to 19) of rock glaciers would remain under the +2 °C threshold, and under 2080s warming, the MAAT at just one rock glacier (range 0 to 3) is projected to remain under +2 °C (Table 1). The relation between MAAT and elevation did not differ substantially between present, 2050s and 2080s climates (estimated lapse rate of ~1 °C/150 m from the WorldClim data, Fig. 3).

## Discussion

We mapped the current and likely future distribution of mountain permafrost across the Bolivian Andes (Fig. 2; Table 1). The sparse and isolated nature of Bolivian permafrost is clear (Fig. 2a), especially when compared to the spatially extensive permafrost along the Chilean/Argentinean Andes between 27° and 35° S (Gruber 2012). This supports our previous work (Rangecroft et al. 2015), which established that Bolivian rock glaciers are less abundant than those of the Chilean Andes (Azócar and Brenning 2010) and the Argentinean Andes (Perucca and Esper Angillieri 2011). The limited distribution of areas with MAATs below 0 °C in the Bolivian Andes likely contributes towards the low frequency of rock glaciers in this region.

**Fig. 4:**
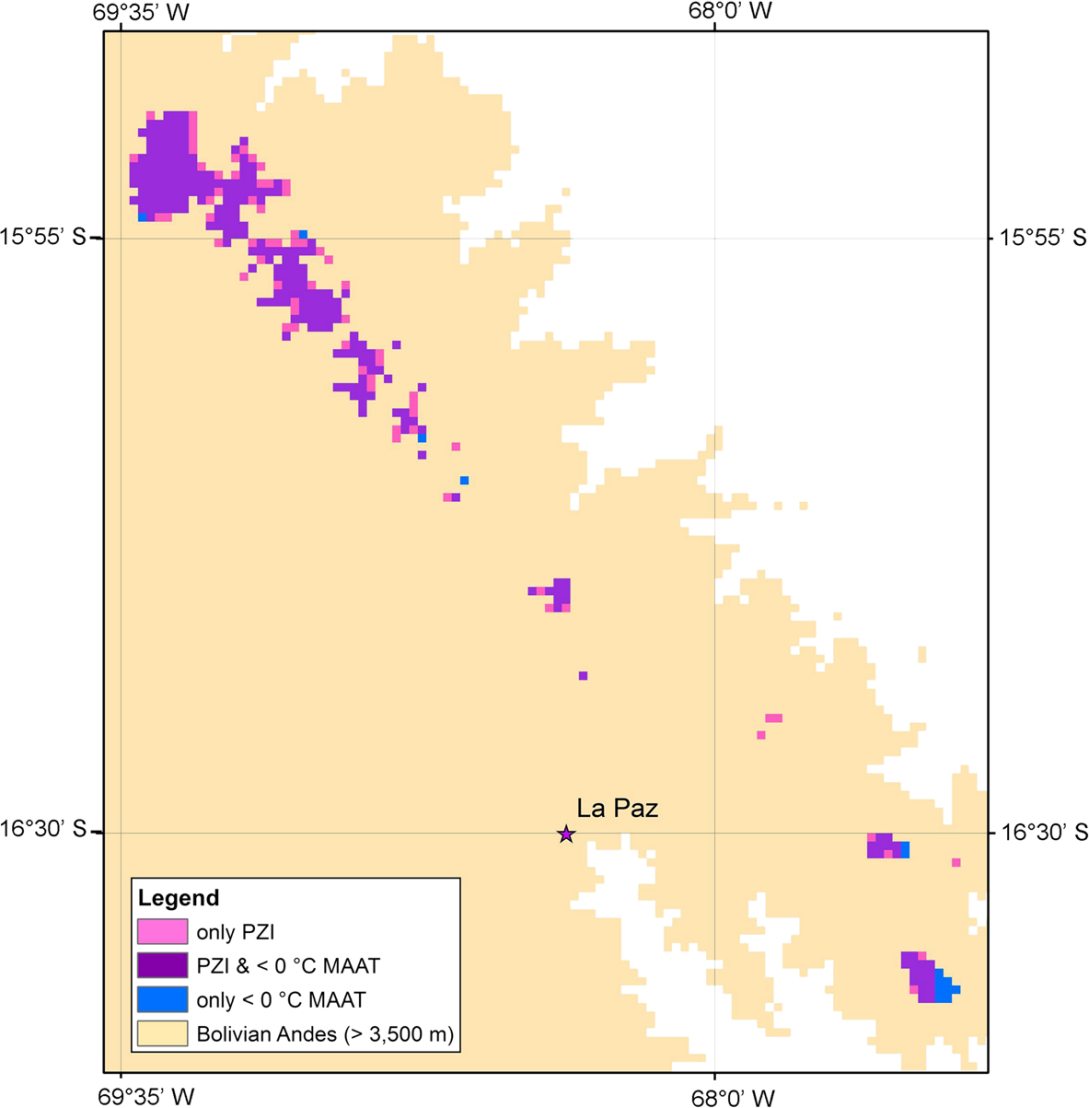
An example from the Cordillera Real region of the spatial comparison of Gruber’s (2012) Permafrost Zonation Index (pink coloration) with our MAAT 0 °C isotherm mapping (blue), highlighting the high level of agreement (purple). Figure was generated in ArcGIS 10.1

Permafrost extent is known to be associated with MAAT thresholds (Gruber 2012), but other local factors can also be important, such as topography, aspect, insolation, vegetation, and snow cover (Gruber 2012). These can be difficult to model without fine resolution data. Although permafrost modelling by Bolch et al. (2011) used MAAT and solar radiation to develop a permafrost model for the Tian Shan region, small-scale variability remained very difficult to capture (Buchroithner and Bolch 2014). Furthermore, other variables, including changes in the spatial and temporal distribution of precipitation, influence permafrost extent and could affect rock glacier development and persistence (Haeberli et al. 1993). Precipitation projections were not included in this analysis due to large uncertainties surrounding the direction and magnitude of future change, unlike the consensus between models regarding the direction (and sometimes magnitude) of temperature change (IPCC 2013). To test the sensitivity of our conclusions to other potential controls of permafrost extent, we conducted a brief comparison between our results and Gruber’s (2012) Permafrost Zonation Index (PZI) (Fig. 4). Despite our relatively simple approach to mapping MAATs, we found strong agreement between the two approaches (Fig. 4). We thus have confidence that our estimates of spatial extent are realistic for the areas we have modelled.

Rock glaciers were found to be strongly associated with the 0 °C isotherm along the Cordillera Occidental in Bolivia. This, along with the relatively homogenous temperature conditions throughout the year in Bolivia (Rabatel et al. 2013), justifies the use of the 0 °C isotherm as a proxy for permafrost extents in this region. It is these similar summer and winter conditions in the tropical Andes which make it very different from the majority of existing mountain permafrost literature. In the European Alps for example, it is thought that active rock glaciers require a MAAT of less than −1 or −2 °C (Brenning 2005), whereas the average MAAT at rock glacier locations in the Bolivian Andes was found to be +0.1 °C. Relatively high rock glacier MAATs were found in the Cordillera Real, with an average MAAT of +1.7 °C and range of +0.4 to +3 °C (Fig. 3). Similar (above zero) rock glacier temperatures have also been observed in the Chilean and Argentinean Andes (e.g. Trombotto et al. 1997; Brenning 2005). Warmer MAATs that remain suitable for permafrost is likely a consequence of local topographic and/or microclimatic factors combining to preserve ice in otherwise climatically unfavourable locations (Bonnaventure and Lewkowicz 2011). This can lead to persistent areas of snow and ice cover despite warmer temperatures in the wider atmosphere. To confirm this effect, direct, in situ field measurements will be required, to confirm this ‘thermostatic’ effect.

Whilst we argue that rising temperatures will reduce the area suitable for rock glaciers (Fig. 2) and result in permafrost degradation (Kellerer-Pirklbauer et al. 2011), other factors could play a role in rock glacier formation. For instance, it is possible that debris fluxes which supply rock glaciers could increase in a warmer climate. Frost shattering is controlled by freeze-thaw cycles, with an elevation range termed the ‘talus window’ (Hales & Roering 2007), and an increase in talus production could increase the debris supply to existing rock glaciers and produce new ones at sites where ice is available. Conversely, rock glaciers are normally located in the upper part of the talus window, and with the talus window expected to move towards higher elevations with a changing climate, a reduction in the area available for talus production and rock glacier supply is to be expected. Just as importantly, rock glaciers are known to respond slowly to changes in temperature (Janke 2005), and so a time lag between observed temperature increase and rock glacier response is expected. However, there is little information on such lag times, and due to the complexity of the energy balance at glacier and permafrost surfaces, potential future changes can only be roughly estimated (Haeberli and Beniston 1998). Future research should target these knowledge gaps and focus on permafrost and rock glacier ice response to climate change.

There is often a complex relation between modelled temperature increase and the response of cryospheric features and landforms, including permafrost and rock glaciers. Yet, the direction of the change projected clearly demonstrates a future reduction in permafrost extents and loss of currently active rock glaciers and suitable areas for rock glacier development (Table 1; Fig. 2). The hydrological significance of rock glaciers in the region and their recession is currently uncertain or unknown, and existing work only begins to address these gaps (e.g. Rangecroft et al. 2015). It will be critical to establish the impact of permafrost and rock glacier recession on water supply for large urban centres such as El Alto and La Paz, especially as they lie in a region already suffering acute water scarcity (Rangecroft et al. 2013). As one of South America’s fastest growing cities, water stresses are expected to be amplified in La Paz by glacier recession, population increase, and projected increases in rural-to-urban migration driven by climate change and westernization of lifestyles (Vanham and Rauch 2010; Buytaert and De Bièvre 2012; Rangecroft et al. 2013). These projected changes in demand combined with changes to water supplies are expected to have critical negative impacts on water security, affecting environmental, economic and social systems (Bradley et al. 2006; Rangecroft et al. 2013).

Furthermore, the implications of these projected changes extend beyond water resources; permafrost changes also affect natural hazards such as slope instability, rockfalls, and glacial lake outburst floods (GLOFs) (Haeberli and Beniston 1998; IPCC 2007). On slopes steeper than 25° to 30°, decreased stability can develop in freshly exposed or thawing nonconsolidated sediments (Haeberli and Beniston 1998). As a result, there is a growing need to integrate climate change adaptation with disaster risk reduction, particularly in glaciated mountain regions, especially as glacial hazards threaten societies in these regions (e.g. GLOFs in Peru, Carey et al. 2012).

## Conclusion

There is a clear need to address the important gaps in knowledge and literature on the Southern Hemisphere’s cryosphere. Given the sensitivity and vulnerability of countries such as Bolivia to climate change, this work highlights the susceptibility of water supply strategies to climate change in such regions. The results here suggest a dramatic loss of permafrost extent in response to projected 21st century warming, representing a reduction of high mountain water storage. With an expected increasing demand for water supplies in Andean cities such as La Paz and surrounding regions, further research on the impacts of projected climatic change on the cryosphere and water resources is essential for future planning and mitigation. Specifically, further research on the Andean permafrost dynamics is required, combined with a better understanding of the relation between permafrost and climate change to improve the anticipation of water supply shortages in the future and natural hazards.
